# The Hygiene of Modern Armies in the Field

**Published:** 1919-01-25

**Authors:** 


### THE HYGIENE OF MODERN ARMIES IN THE FIELD.

Of all the problems with which the Medical Services are confronted during war, by far the most important is the maintenance of the health of the troops in the field. In spite of the enormous increase in importance of the artillery and mechanical devices of all kinds (including gas), the success of operations still depends ultimately on the ability of a commander to throw a preponderating number of "effective" troops into the scale at a given moment. If the health of the troops is not maintained, they cease to be effective. Not only is every sick man an absolute loss to his unit, but also he increases the work of the medical personnel and adds to the burden of transport.

Hygiene, or the science of the maintenance of health, is therefore a matter which affects everyone, from the Commander-in-Chief downwards. The General Staff know that, if the number of sick men increases, there will be fewer fighting troops to carry out their plans. The Administrative Staff know, similarly, that there will be fewer men available for feeding, moving, and bringing ammunition and other stores to the fighting troops, who, in their turn, will suffer. There are of course other important factors to be considered in this connection—such as the supply of reinforcements and the "Sick Wastage," that is, the number of men who are too ill to be treated in field-hospitals, and have to be sent to the base, or to England, for treatment. These are, for the time being, a complete loss to the armies in the field. But the rise, or fall, of the "sick rate" is the truest index of an army's capacity for success, and the wise General keeps a very close watch upon it.

### The Sick Rate.

The number of men who are admitted to hospital on any given day, for reasons other than wounds, compared with the total number of men in the unit, is the sick rate of that unit. For example, if a battalion is composed of 1,000 officers and men, and of these four are sent into hospital suffering from disease on December 1st, the sick rate for that day is .4%. The Chief Medical Officer in every division, corps, and army gets the figures from all the medical units under his charge, and, knowing the total number of troops, he can work out this "sick rate" and keep the General in command informed as to the condition of his troops. If the rate in any unit rises above a certain point, he, or one of his staff, makes enquiries as to the reason, and takes steps to remove the cause if possible. These figures and calculations may seem to the uninitiated to be mere "red tape," but it is obvious that without such information a General cannot know what forces are available for fighting battles.

### Factors in Maintaining Health.

Just as there are many different circumstances which go to the making of a healthy man, so there are many factors also which have to be considered in order to keep men healthy. Much of the spade work is done before men go overseas, by the carefully graduated training of recruits, the weeding out of those unfit for active service, and special measures—such as vaccination and inoculation—which protect soldiers against those diseases from which an army in the field is peculiarly liable to suffer. The special measures required in the field may be best described under the following heads:

1. *Food.*—Both the quantity and quality of the food issued to soldiers have an important bearing upon their health. There must be enough of the principal ingredients in a diet, *viz.*, meat, fat, bread and sugar, to enable men to live a life of hard work. The diet must be sufficiently varied to avoid the distaste and digestive troubles inseparable from lack of change. Fresh vegetables must be included so as to avoid the danger of scurvy. It has to be remembered also that all food may have to be carried hundreds, or even thousands, of miles to an army in the field, so that all unnecessary articles of diet have to be rigidly excluded, and, where two articles are of equal nutritious value, the least bulky of the two must be chosen, for the same reason.

Another very important point is the time at which meals are given to the men; this must be arranged to interfere as little as possible with their work. The advantage of having hot meals, both from the point of view of being more digestible, and also because of the increased comfort derived from them, more than compensates for the difficulty, often the very great difficulty, of preparing hot food and conveying it to men in the firing line. As an instance of this it may be mentioned that, in trench warfare, the least sign of smoke from a cookhouse anywhere near the front is apt to draw shell fire, and communication with front line trenches is often only possible at night for the same reason. When troops are on the move, the food is prepared in travelling kitchens, drawn by horses, which cook as they go along. But here again, when it is remembered that a division on the march occupies about fifteen miles of road, it will be realised that it is no easy matter to have a hot meal ready for men at the time when they want it most—at the end of the day's march. While the medical authorities decide on the quantity and kind of food which is to be issued, the actual duty of supplying it belongs to the Royal Army Service Corps.

2. *Water.*—Water is a necessity of life, not only as an article of diet, but also because it has to be evaporated from the surface of the body in order to maintain an even temperature and prevent the body from becoming overheated. It is also required for cooking, and for washing. The provision of water is one of the duties of the Royal Engineers, but the medical authorities have to decide if it is fit for use and, if not, on the measures which must be taken to purify it. Much

**Hygiene of Modern Armies in the Field**—(*cont.*).

of the water available for an army in the field is unfit for use, either from natural causes, or because it has been deliberately fouled by the enemy. The two methods used for purifying water are boiling and the addition of chemicals. The former is often impossible to carry out on a large scale, so that the latter is usually employed. As the effects of drinking impure water may be the outbreak of such diseases as typhoid fever, dysentery, and cholera, it is clear that this matter is one which has to be most carefully and thoroughly gone into. The least breakdown in the arrangements may be the cause of much illness, and even many deaths.

3. *Clothing.*—The supply of suitable clothing and boots to soldiers is very necessary if their health is to be maintained on active service. While the medical service advise on the question of suitability, and such matters as the necessity for the issue of extra blankets, &c., the clothes and boots are actually supplied by the Royal Army Ordnance Corps.

4. *Washing.*—In order to keep healthy and free from skin diseases, regular washing of the surface of the body is necessary. This is no easy matter to arrange for large bodies of men living under conditions such as obtain in modern warfare. Bath houses, generally fitted with spray baths, are provided at some distance behind the front line, and, when troops are "out of the line," every opportunity is taken of enabling men to have regular baths. At most bath houses they are supplied with clean underclothes, and the dirty clothes are sent off to large steam laundries in towns some distance away.

During the winter when men are liable to get "trench foot" as the result of exposure to wet and cold, hygienic precautions have to be taken to guard against this evil. Chiropodists attend at the baths, and the men's feet are massaged with special preparations.

5. *Anti-Fly Measures.*—As it is known that flies spread infection, special precautions have to be taken to prevent their increase. All refuse is carefully collected and burned or buried, and all horse manure is so treated as to prevent its being used as a breeding place for flies. The measures taken for the disposal of all waste matter are often peculiarly difficult, both on account of its bulk and because of the large amount of labour required for its collection, removal, and incineration or burial. The material required for building incinerators is often hard to obtain, though full use is made of all sorts of makeshifts—such as tins filled with earth, sods of earth, broken bricks, corrugated iron &c.

In addition to the above there are many other subjects which have to be studied to keep a *mens sana in corpore sano.* "BLUE TABS."

